# Editorial: Application of gene editing in pathology dissection of neurodegenerative diseases

**DOI:** 10.3389/fnins.2022.1092176

**Published:** 2023-01-05

**Authors:** Sen Yan, Xingshun Xu, Fangfang Qi, Xing Guo, Junhong Luo, Yujing Li

**Affiliations:** ^1^Guangdong Key Laboratory of Non-human Primate Research, Guangdong-Hongkong-Macau Institute of Central Nervous System (CNS) Regeneration, Jinan University, Guangzhou, China; ^2^The Institute of Neuroscience, Soochow University, Suzhou, China; ^3^Department of Anatomy and Neurobiology, Guangdong Province Key Laboratory of Brain Function and Disease, Zhongshan School of Medicine, Sun Yat-sen University, Guangzhou, China; ^4^Department of Neurobiology, School of Basic Medical Sciences, Nanjing Medical University, Nanjing, Jiangsu, China; ^5^Department of Systems Biomedical Sciences, School of Medicine, Jinan University, Guangzhou, China; ^6^Department of Human Genetics, Emory University, Atlanta, GA, United States

**Keywords:** gene, editing, CRISPR, neurodegeneration, animal models, cell model, pathogenesis, physiology

Neurodegenerative disorders (NDs) refer to a subset of neurological diseases prevalent among the aging population. NDs are mainly characterized by progressive neuronal loss in the central nervous system (CNS) and include diseases such as Alzheimer's disease (AD), Huntington's disease (HD), Parkinson's disease (PD), Amyotrophic Lateral Sclerosis (ALS), and so on. Patients with such disorders suffer from devitalizing memory loss and impaired motor coordination. Although the real cause of NDs remains largely to be discovered, it is fully appreciated that both environmental and genetic/epigenetic factors contribute to the pathophysiology of these disorders. Indeed, significant discoveries have been made in unveiling the cellular and genetic mysteries behind the physiopathology of NDs in the recent decade thanks to the application of state-of-the-art biotechnology-based approaches, including single-cell sequencing, and the CRISPR-CAS based genome editing technology. Given that animal and *in vitro* cell models contribute significantly to the pathophysiological study of human diseases, genome editing based generation of specific ND animal and organoids models usher in a new era to investigate the pathogenesis of NDs and, ultimately, clinical therapy.

Since the invention of CRISPR-CAS gene-editing, pivotal breakthroughs have been made in generation of genome-edited animal/organoids models of NDs. Even though some drawbacks remain that affect the efficiency, quality, and application of these models, previous achievements in gene editing based disease models have provided insight into the molecular mechanisms underlying NDs and inform future studies of research avenues worthy of pursuit.

This Research Topic is primarily focused on the generation of new ND models through gene editing. The aim of this Research Topic is to provide a broad overview of the current research on better simulation of the ND phenotype and exploration of its pathological characteristics and pathogenesis, as well as drug screening and validation of potential drugs using gene-edited ND models. A major emerging theme in this field is the potential translation from ND models to future clinical therapy.

It is well-documented that metabolic disorders are associated with neurodegenerative diseases such as AD, epilepsy, and Leigh's syndrome. However, limited information is available on the molecular mechanisms. To investigate the contribution of PDHA1 deficiency to neurodegenerative disorders, Chen et al., generated the CRISPR-CAS based hippocampus-specific Pdha1 KO mice. It was found that Pdha1 deficiency led to accumulation and abnormal transport of lactate in the hippocampus, ultrastructural alteration of hippocampal neurons, and damage of the spatial memory, in addition to inhibition of the cAMP/PKA/CREB pathway. Therefore, the authors suggest that the lactate accumulation caused by PDHA1 deficiency may impair cognitive function through inhibition of the cAMP/PKA/CREB pathway in hippocampus.

It is known that ubiquitin–proteasome system (UPS) contributes to the perioperative neurocognitive disorders (PND) caused by isoflurane exposure, but the related molecular mechanism remains to be understood. To explore the potential mechanisms, Xu et al., generated the ubiquitin E3 ligase protein carboxyl-terminus of Hsc70-interacting protein (CHIP) knock-down N2a cells. By comparing the consistency in alteration of synapsin expression and phosphorylation between the CHIP knock-down N2a cells and the isoflurane-exposed aged mouse, the authors concluded that synaptic degeneration is caused by the reduced expression of CHIP, and could contribute to pathogenesis of PND in exposure to isoflurane.

Genetically, relatively few genes have been identified that contribute to the pathology of AD. To further identify additional relevant genes and validate the genes predominantly involved in AD pathogenesis, Zhang et al., bioinformatically analyzed 1,153 aging and senescence-associated genes. Based on the results from the bioinformatic analysis, five aging-related differential expression genes (ARDEGs) were chosen for further validation by using molecular comparison between control and AD populations. Based on this study, the authors suggested that four ARDEGs, including PDGFRB, PLOD1, MAP4K4, and NFKBIA are involved in aging, cellular senescence, and that Ras protein signal transduction regulation could potentially serve as a novel biomarker for AD diagnosis and progression. More importantly, this study highlights how aging could function as one of the key risk factors for AD pathogenesis.

In addition to the genetic basis of NDs, the role of epigenetic regulation in NDs has started to receive more attention in the recent decade. In this Research Topic, instead of utilizing the application of genome-editing in NDs, Zhu et al., investigated alterations of the m6A RNA methylation in children with neurological disorders in response to enterovirus infection. It was found that there are significant changes in RNA m6A methylation patterns and there is an enrichment of m6A in genes involved in the oxidative phosphorylation pathway, PD, and additional metabolic pathways were observed in the patients relative to the control children group.

As the articles of this Research Topic state, the lack of appropriate ND models has become a bottleneck for pathological investigation, whereas novel genome editing technology provides a powerful and versatile tool for generation of *in vivo* and *in vitro* models. The genome editing based animal models and human organoids are essential for the investigation of physiopathology, and drug screening as well as the development of clinical therapy for NDs. Given the discrepancy in genetic, anatomic, and physiological aspects caused by evolution levels between mouse and human, we hope that future work will focus on gene editing based human brain organoids. Relative to animal models, the human brain organoids could provide more complete models of ND pathogenesis to make the pathological study easily translatable to clinical applications, particularly toward personalized medicine.

In addition to research articles, two review articles by independent groups (Lu et al. and Zhou et al. groups) highlight recent advances in the application of CRISPR/Cas9 in AD. The authors from both groups discussed the challenges and potential strategies regarding the generation of AD animal and cell models, identification of pathogenic genes, drug screening, target therapy, and cellular reprogramming. These review articles provide important scientific information in a convenient manner that is targeted toward readers with a strong interest in closely related research fields.

## Author contributions

SY and YL drafted the editorial. SY and YL revised the editorial with contributions from all authors. All authors approved the final version.

